# A Case of Human Immunodeficiency Virus (HIV) Infection Leading to Acquired Immunodeficiency Syndrome (AIDS)-Related Pneumocystis Pneumonia

**DOI:** 10.7759/cureus.103602

**Published:** 2026-02-14

**Authors:** Mahika Shetty, Jyothi R Patri

**Affiliations:** 1 Research, Drexel University College of Medicine, Philadelphia, USA; 2 Family and Lifestyle Medicine, Heritage Valley Family Medicine Residency Program, Beaver Falls, USA

**Keywords:** aids, haart therapy, hiv, immunosuppression, pneumocystic pneumonia, primary care physicians, severe cd4 depletion, universal hiv screening

## Abstract

Pneumocystis pneumonia (PCP) is a serious pulmonary infection that frequently occurs in individuals with advanced human immunodeficiency virus (HIV) infection or acquired immunodeficiency syndrome (AIDS), especially when profound immunosuppression is present. Delayed HIV diagnosis may lead to progression to AIDS and subsequent development of opportunistic infections, including PCP.

A 39-year-old woman presented to the emergency department with a two-week history of persistent cough, chest pain, and shortness of breath. She reported intermittent illness over the preceding five months, along with an unintentional 40-pound weight loss. On admission, she was febrile, tachypneic, tachycardic, and hypoxic. Chest computed tomography revealed bilateral pneumonia. Physical examination identified white oral plaques suggestive of fungal infection. Laboratory evaluation demonstrated a CD4 count of 2 cells/mm³. Bronchoscopy confirmed the diagnosis of PCP in the context of AIDS secondary to HIV infection.

The patient received treatment with trimethoprim-sulfamethoxazole, fluconazole, and high-dose prednisone for hypoxia, in addition to initiation of highly active antiretroviral therapy. Counseling was provided regarding her diagnosis, medication adherence, and the necessity of follow-up care. She was discharged with instructions for close outpatient follow-up with a primary care physician and referral to an HIV specialist.

This case highlights the critical importance of routine HIV screening, since delayed diagnosis can result in advanced disease, increased morbidity, and greater healthcare expenditures.

## Introduction

Pneumocystis pneumonia (PCP) is a fungal opportunistic infection caused by the yeast-like fungus *Pneumocystis jirovecii*. This organism is commonly present in the lungs of healthy individuals but becomes active as an opportunistic infection when both cellular and humoral immunity are compromised, particularly in those with human immunodeficiency virus (HIV) infection and CD4 counts below 200 cells/mm³ [1,2]. The trophic forms of *Pneumocystis* attach to the alveoli upon inhalation [1,2]. Studies have shown that approximately 1.1 million people in the United States are currently living with HIV, and only 15% of them are aware of their diagnosis, which significantly delays timely treatment [3]. Prior to the widespread availability of antiretroviral therapy, PCP was among the most common AIDS-defining illnesses and continues to represent a frequent initial presentation of previously undiagnosed HIV infection, especially in individuals without routine access to preventive healthcare or screening [2,3]. The susceptibility to PCP in advanced HIV is largely driven by progressive CD4 T-lymphocyte depletion, resulting in impaired alveolar macrophage activation and ineffective clearance of *Pneumocystis* organisms from the lungs [1,4]. Understanding the immunologic basis of HIV progression, including CD4 and CD8 T-cell dynamics, is essential for recognizing why patients with profound CD4 depletion are particularly susceptible to opportunistic infections such as PCP.

## Case presentation

A 39-year-old woman with a history of gastroesophageal reflux disease (GERD) and controlled asthma presented to the emergency department with a two-week history of a productive cough, pleuritic chest pain, and dyspnea. She reported thick yellow-green sputum production, exertional dyspnea progressing to dyspnea at rest, and severe coughing episodes associated with headaches. Additional symptoms included diarrhea, nausea, and occasional vomiting. Over the preceding four to five months, she experienced recurrent respiratory illnesses, profound fatigue, chronic diarrhea, nausea, intermittent vomiting, and an unintentional weight loss of approximately 40 pounds. She denied recent travel, known tuberculosis exposure, or intravenous drug use. Her symptoms did not improve despite over-the-counter medications and prescribed inhaler therapy, prompting hospital evaluation.

The patient's medical history was notable for homelessness lasting over one year, resulting in limited access to health care. She contacted her sister for assistance when her illness worsened, and she was unable to care for herself. Although her sister encouraged her to seek medical attention, the patient was reluctant due to concerns regarding insurance coverage. There was no documented history of sexually transmitted infections.

Upon admission, the patient met the criteria for sepsis. Initial laboratory assessments included complete blood count (CBC), comprehensive metabolic panel (CMP), troponin, basic metabolic panel (BMP), liver enzymes, procalcitonin, and lactic acid levels, and were all within normal limits (see Table 1) [3]. Urinalysis showed trace leukocyte esterase with negative blood and nitrites. Respiratory viral panel, including COVID-19, urine drug screen, and blood cultures, were negative.

**Table 1:** Admission vital signs and laboratory findings This table summarizes the patient’s laboratory results after admission to the hospital. The results highlight the severity of her immunosuppression, as her CD4 count was 2 cells/mm³ [3]. CBC, complete blood count; CD4, cluster of differentiation 4; CMP, comprehensive metabolic panel; COVID, coronavirus disease; HIV, human immunodeficiency virus; RNA, ribonucleic acid; PCR, polymerase chain reaction

	Test conducted	Results	Reference range
Vital signs	Temperature	100.9°F	<100.8°F
	Heart rate	127 beats per minute	60-100 beats per minute
	Respiratory rate	45 breaths/minute	12-20 breaths/minute
	Oxygen saturation (room air)	87%	95-100%
Laboratory findings	CD4 count	2 cells/mm³	600-1000 cells/mm³
	HIV-1 RNA PCR	692,088 copies/mL	Not detected
	CBC	Normal	NA
	CMP	Normal	NA
	Lactic acid	Normal	<2.0 mmol/L
	Troponin	Normal	0-0.04 ng/mL
	Pro-calcitonin	Normal	<0.10 ng/mL
	Beta-D-glucan	Positive	Negative
	Respiratory viral panel (including COVID)	Negative	Negative
	Blood cultures	Negative	NA

Physical examination revealed an acutely ill-appearing patient with marked conversational dyspnea and accessory muscle use. Oral examination demonstrated white plaques and ulcerations involving the tongue and soft palate, consistent with oral candidiasis. Lung auscultation revealed diffuse bilateral crackles without focal consolidation.

**Figure 1:**
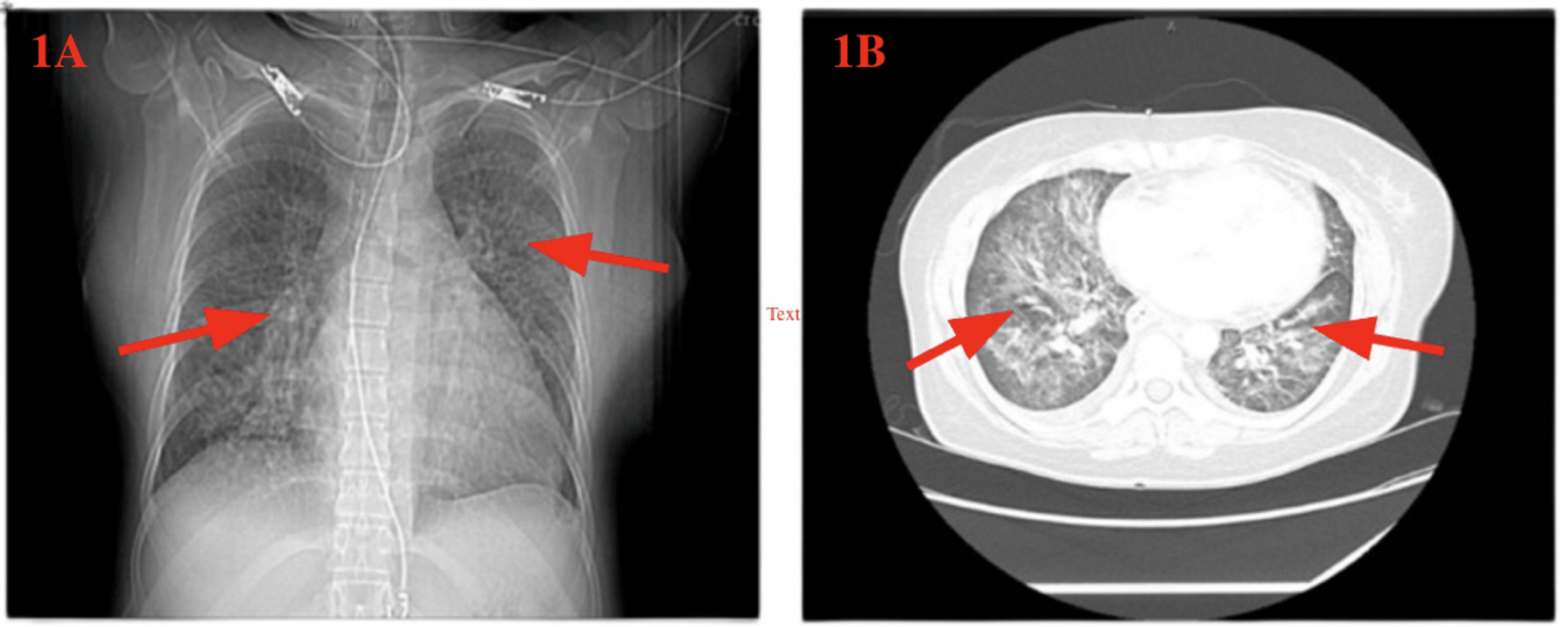
Lung imaging findings in Pneumocystis jirovecii pneumonia (A) Chest X-ray demonstrating hazy, patchy bilateral pulmonary opacities. (B) Chest CT scan demonstrating widespread ground-glass opacities in both lungs. These findings are suggestive of *Pneumocystis jirovecii* pneumonia in a patient with a very low CD4 count. CT, computed tomography; CD4, cluster of differentiation 4

HIV-1 ribonucleic acid (RNA) polymerase chain reaction (PCR) testing revealed 692,088 copies/mL, and the absolute CD4 count was noted to be 2 cells/mm³. The rest of the STD testing was unremarkable. Cocci antibodies, blood, and urine culture were normal. Autoimmune testing was normal; however, the inflammatory markers were elevated. QuantiFERON-TB Gold testing was indeterminate. Beta-D-glucan testing was positive. A chest X-ray showed bilateral pulmonary infiltrates, as seen in Figure 1, and a CT scan of the chest showed ground-glass opacities in both lungs, consistent with diffuse pneumonitis rather than viral pneumonia, as seen in Figure 1.

Given the patient’s hypoxia, chronic systemic symptoms, significant weight loss, and bilateral pulmonary infiltrates, the differential diagnosis included atypical bacterial pneumonia, viral pneumonitis, pulmonary tuberculosis, fungal pneumonia, pulmonary embolism, and *P. jirovecii* pneumonia in the setting of suspected immunosuppression.

Owing to the presence of severe hypoxia, diffuse ground-glass opacities on chest imaging, a positive beta-D-glucan assay, and profound CD4 depletion, bronchoscopy with bronchoalveolar lavage was pursued to establish a definitive microbiologic diagnosis, consistent with recommended diagnostic approaches for suspected PCP in patients with advanced HIV infection. Bronchoscopy showed *Candida albicans* and mixed respiratory flora. Bronchoalveolar lavage PCR was positive for *P. jirovecii*, confirming the diagnosis of AIDS due to HIV complicated by PCP.

An infectious disease (ID) consultation was obtained, and the patient received high-dose trimethoprim-sulfamethoxazole, fluconazole, and prednisone as recommended. High-dose steroids were administered due to severe hypoxia. Subsequently, highly active antiretroviral therapy (HAART) was initiated under ID supervision and continued after discharge. An urgent referral to an HIV specialist was also requested for close follow-up. Currently, the patient's condition has shown improvement, and she remains compliant with her medications and follow-up appointments.

## Discussion

HIV is a single-stranded genomic RNA retrovirus that attacks the CD4 (T-helper) cells. The normal range of CD4 cells is 600-1000 cells/mm³, and this level decreases by 50-100 cells per year in an untreated person. Progressive depletion of CD4 cells takes about five to 10 years before patients manifest clinical symptoms. HIV itself may not cause illness and death like any other chronic disease. The Centers for Disease Control and Prevention (CDC) defines an HIV-positive condition as AIDS when either the CD4 count drops below 200 cells/mm³ or a person develops an AIDS-defining illness, including opportunistic infections, malignancies, or neurological diseases, regardless of their CD4 count [5].

CD4 T-lymphocytes play a central role in coordinating cell-mediated immunity, and their progressive depletion in untreated HIV infection results in impaired macrophage activation and diminished clearance of opportunistic pathogens such as P. jirovecii [4,5]. Although CD8 T-cell counts may initially increase during early stages of HIV infection, they are insufficient to compensate for advanced CD4 loss, leading to vulnerability to AIDS-defining opportunistic infections, including PCP [4,6].

**Figure 2:**
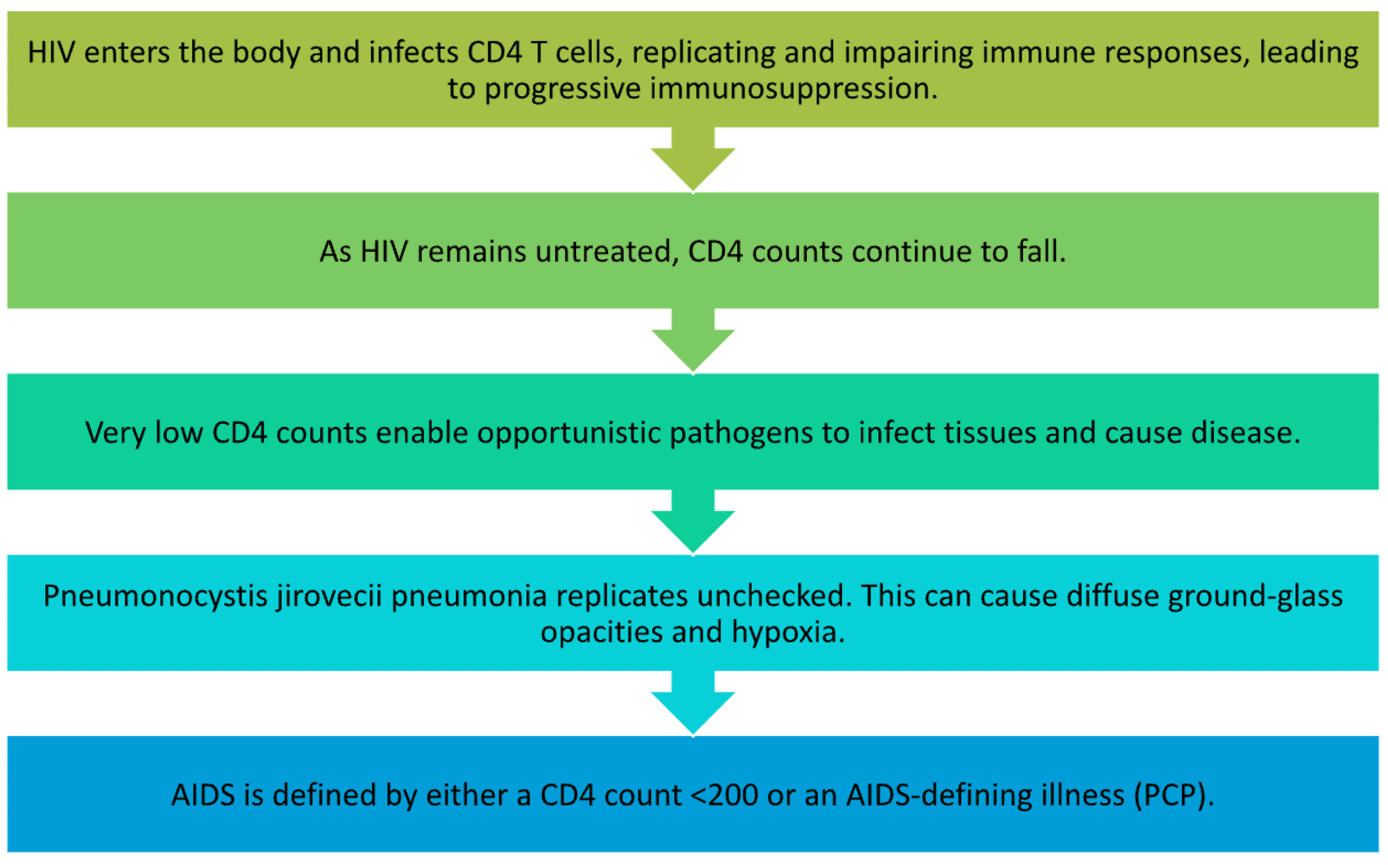
Progression from HIV infection to AIDS and development of PCP This figure outlines the progression from initial HIV infection to severe CD4 depletion, resulting in immunosuppression. At critically low CD4 levels, uncontrolled *Pneumocystis jirovecii* proliferation leads to pneumonia, marking the transition to AIDS. AIDS, acquired immunodeficiency syndrome; CD4, cluster of differentiation 4; HIV, human immunodeficiency virus; PCP, pneumocystis pneumonia Source: Flowchart created based on data synthesized from [4].

HIV remains a serious public health issue. Studies estimate that one in seven people with HIV is unaware that they are infected with the virus [3]. The U.S. Preventive Services Task Force (USPSTF) recommends universal HIV screening for all ages between 15 and 65 during annual physicals, while pregnant women need annual screening or more frequent screening [6]. Figure 2 illustrates the disease pathology in the absence of routine HIV screening.

Primary care physicians play an essential role in optimizing routine HIV screening during annual physical appointments and prescribing pre-exposure prophylaxis (PrEP) and post-exposure prophylaxis (PEP) when necessary. Studies indicate that HAART can significantly reduce HIV-associated morbidity and mortality, decrease the transmission of the virus, and may even reduce the risk of serious non-AIDS-related diseases.

**Figure 3:**
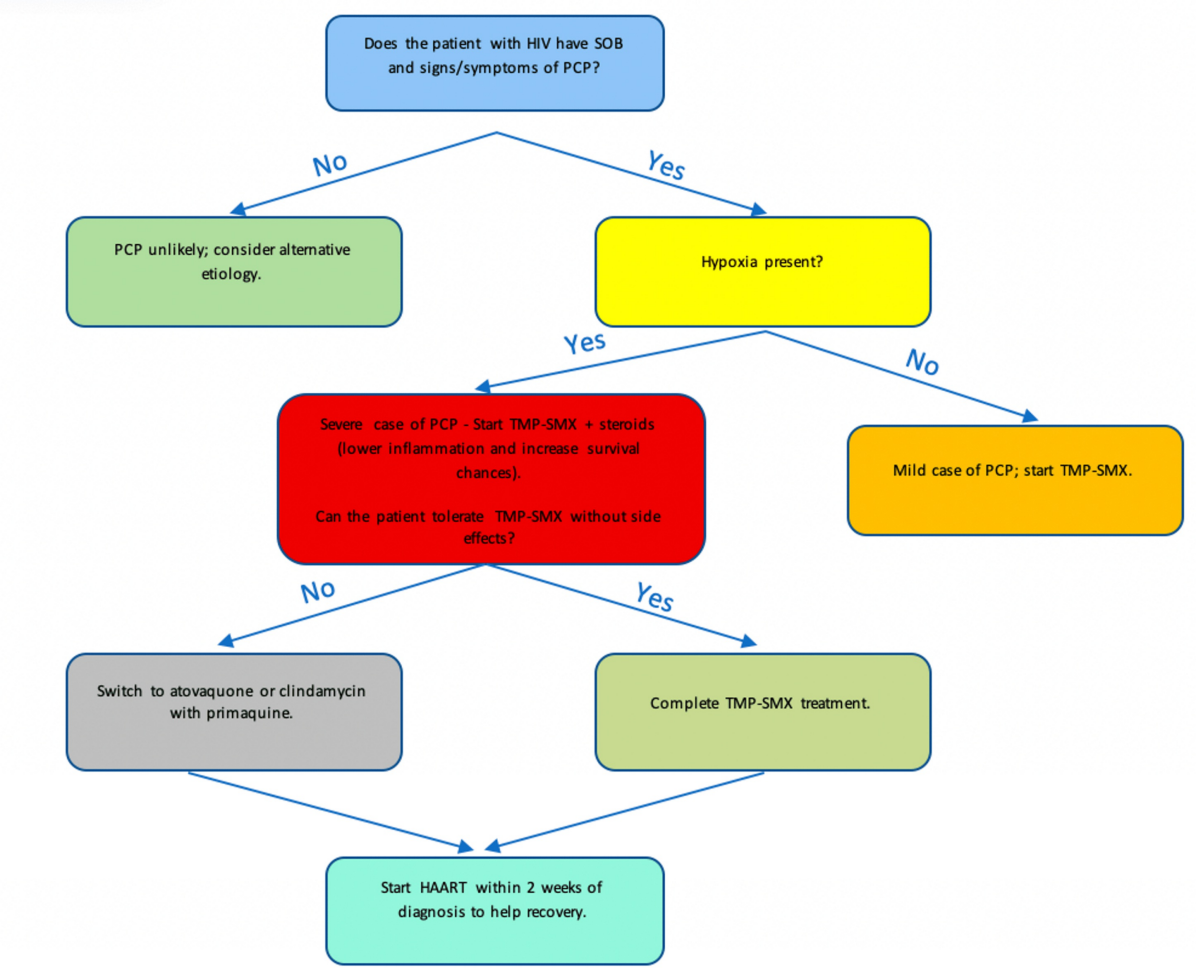
Flowchart synthesized for PCP treatment in HIV This flowchart outlines the diagnostic and treatment protocols for PCP in patients with HIV. It also highlights the importance of HAART in combating this disease. HAART, highly active antiretroviral therapy; HIV, human immunodeficiency virus; PCP, pneumocystis pneumonia; TMP-SMX, trimethoprim-sulfamethoxazole; SOB, shortness of breath Source: Flowchart created based on data synthesized from [7-9].

This case is notable because the patient's initial presentation revealed advanced HIV infection with a CD4 count of 2 cells/mm³. Most patients develop symptoms before the CD4 count reaches this critical level, and such a low count at initial presentation and diagnosis is uncommon. In this patient, earlier HIV screening could have identified the disease, helping with prompt initiation of appropriate treatment, given the fact that the patient had experienced four to five months of prolonged illness before the diagnosis. As Figure 3 depicts, regardless of the treatment prescribed, studies strongly recommend the initiation of HAART within two weeks of PCP treatment as it reduces the risk of disease progression [7-9].

Early diagnosis prevents disease progression and the development of complications such as respiratory failure requiring ventilatory support and pneumothorax resulting from alveolar injury [10]. Delayed diagnosis may also result in neurological complications, blindness associated with cytomegalovirus retinitis, or chronic pulmonary impairment [11]. These risks highlight the importance of routine HIV screening by primary care physicians, regardless of the presence of underlying symptoms.

## Conclusions

PCP is a life-threatening, AIDS-defining opportunistic infection and may serve as the initial presentation of undiagnosed HIV. The patient's critically low CD4 count suggests a progressive immunocompromised state before seeking medical attention. Prompt diagnosis and treatment with TMP-SMX, steroids for hypoxia, and early initiation of antiretroviral therapy can prevent the progression to advanced disease and complications. This case emphasizes the need for routine HIV screening and maintaining a high index of suspicion for systemic symptoms such as weight loss, chronic cough, or frequent infections. Early detection of HIV results in prompt management and prevents complications such as PCP, thereby reducing the risk of progression to AIDS.
